# Office Hysteroscopy. An operative gold standard technique and an important contribution to Patient Safety

**DOI:** 10.1007/s10397-015-0926-0

**Published:** 2016-02-04

**Authors:** J. Mairos, P. Di Martino

**Affiliations:** Department of Gynecology and Obstetrics, Hospital das Forças Armadas - Pólo de Lisboa, Azinhaga dos Ulmeiros, 1649-020 Lisbon, Portugal

**Keywords:** Office Hysteroscopy, Patient Safety, Health Quality

## Abstract

According to World Health Organization (WHO), about 1 out of 10 hospitalized patients suffers an adverse event, in developed countries, being an adverse event an injury related to medical management, in contrast to complications of disease. These events cause both unnecessary suffering and huge cost to health systems. This issue is so important that WHO has defined it as a global health problem and in 2004 launched the World Alliance for Patient Safety, with the aim to coordinate, disseminate and accelerate improvements in Patient Safety. Office Hysteroscopy (OH), as an independent technique of the hospital circuit, has the ideal conditions to be qualified as the gold standard technique for the surgical treatment of intracavitary uterine pathology. It does not require the use of an operating room, hospital admission and general or locoregional anaesthesia. The appropriate surgical techniques, allied to pain control, allow OH to resolve much more than 90 % of the surgical needs of the intracavitary uterine pathology, thus being an important contribution for Patient Safety.

## Background

The problem of adverse events in health care is known since 1950s. The subject remained largely neglected until early 1990s, when the results of the Harvard Medical Practice Study in 1991 [1, 2] warned of the dimension of the problem in a new economic and social context. Subsequent research in Australia [3], the United Kingdom of Great Britain and Northern Ireland [4] and the USA and in particular the 1999 publication *To err is human: building a safer health system by the Institute of Medicine* [5] provided further data and brought the subject to the top of the policy agenda and the forefront of public debate worldwide. Today, this is a well-known problem and the World Health Organization launched in 2004 the World Alliance for Patient Safety. Some facts mentioned in this Forward Programme in 2004 (http://www.who.int/patientsafety/worldalliance/en/) illustrate the problem:

Estimates show that in developed countries as many as 1 in 10 patients is harmed while receiving hospital care. The harm can be caused by a range of errors or adverse events (http://www.who.int/patientsafety/worldalliance/en/).

Hospital infections affect 14 out of every 100 patients admitted (http://www.who.int/patientsafety/worldalliance/en/).

Surgical care is associated with a considerable risk of complications. Surgical care errors contribute to a significant burden of disease despite the fact that 50 % of complications associated with surgical care are avoidable (http://www.who.int/patientsafety/worldalliance/en/).

Safety studies show that additional hospitalization, litigation costs, infections acquired in hospitals, disability, lost productivity and medical expenses cost some countries as much as US$ 19 billion annually. The economic benefits of improving Patient Safety are therefore compelling (http://www.who.int/patientsafety/worldalliance/en/).

Industries with a perceived higher risk such as the aviation and nuclear industries have a much better safety record than health care. There is 1 in 1,000,000 chances of a traveler being harmed while in an aircraft. In comparison, there is 1 in 300 chances of a patient being harmed during health care (http://www.who.int/patientsafety/worldalliance/en/).

There is now growing recognition that Patient Safety and Quality are a critical dimension of universal health coverage.

A research carried out in three public hospitals in great Lisbon area in 2011 based on a sample of 1669 hospitalized patients and a total of 47,783 hospital admissions showed an Adverse Event’s (AE) incidence of 11.1%, and the most critical places were the room or nursery (49.7 % of the AE) and the operating room (23.9 % of the AE) [6].

Regarding office practice in gynaecological and obstetrics procedures, a task force was convened in 2008 by the American College of Obstetricians and Gynaecologists (ACOG). The primary impetus to creating this task force was the steady migration of surgical procedures to the office that had solely been performed in the hospital or ambulatory surgical center, and this transition began with hysteroscopy in the 1980s [7].

In 2004, Bettochi et al. [8] reported on 4863 operative hysteroscopic procedures performed using a 5.0-mm diameter operative hysteroscope and 5F instruments. The procedures included the removal of cervical and endometrial polyps along with adhesiolysis and repair of “anatomic impediments”, by using a vaginoscopic technique “without analgesics or anaesthesia”, and noted that patients reported little discomfort, although those undergoing removal of endometrial polyps were more likely to experience “moderate” discomfort [9].

So, why would Office Hysteroscopy be such an important contribution for the Patient Safety?

Because, firstly, it is a procedure independent from the hospital circuit, thus avoiding risk of adverse events associated to hospitalization. Secondly, OH has clear advantages before operating room (OR) hysteroscopy, eliminating complications of general or locoregional anaesthesia, being limited only by its surgical ability and pain control.

## Methods

All our OH have been performed by *see and treat* hysteroscopy and by vaginoscopic approach at the Department of Gynaecology and Obstetrics of the Hospital das Forças Armadas in Lisbon.

Preceding all hysteroscopies, patients were evaluated regarding need to treat vaginal pathological discharge, need for local or systemic hormonal treatment and use of misoprostol.

All patients were submitted to pelvic transvaginal ultrasound prior to procedure.

In the day of the hysteroscopy, rectal 10 mg butylscopolamine with 800 mg paracetamol and oral 5 mg diazepam were administered to all patients before the procedure.

Hysteroscopies were performed using rigid 5Fr Bettocchi^®^ hysteroscopes with 30° optic. The distension medium used was saline solution at 37 °C, and the electrodes were Twizzle type bipolar from Versapoint^®^, apart from mechanical instruments. Hysteroscopic anaesthesia (HA), a method of local anaesthesia using an endoscopic needle, was given when requested by the patients, using a Williams Cystoscopic Injection Needle, 22 ga (length 35 cm, point 8 mm) [10]. This method was first described in 2009 by Skensved [11] and allows administration of focal local uterosacral, endocervical or even intracavitary anaesthesia (1 % lidocaine), under hysteroscopic visualization, with no need to interrupt the procedure or use of speculum [10]. When HA was decided, according to specific locations, 1 % lidocaine was injected through the hysteroscope: around 1 cm^3^ per location in the endocervical region, near the internal os or intracavitarily, or 2 cm^3^ at both uterosacral ligaments, if necessary, to a limit of 10 cm^3^ maximum per hysteroscopy.

Procedures were performed by both experienced hysteroscopists and trainees, recorded on DVD, photographed and registered in our database during the global period of February 2012 to April 2014, accordingly to Table 1.

**Table 1:** Characterization of hysteroscopies according to the degree of professional experience

Office Hysteroscopy		Hysteroscopic time (average—min)			Sent to OR
Diagnostic	24.7 %	Total	Experts	Trainees	5.7 %
Operative	75.3 %	24.6	21.7	24.6	

We questioned the patients about pain felt during procedures, by an anonymous survey using a 0–10 pain scale, in which 0 corresponded to no pain and 10 to maximum pain experienced before.

We rely on three retrospective studies conducted in our department: one between April 2011 and April 2014 (330 procedures) that assessed the number of patients sent to the OR, another between May 2010 and March 2012 (207 procedures) that evaluated effectiveness of HA and another between January 2010 and December 2012 (230 procedures) that evaluated the dimensions of the excised masses in OH.

## Findings

In our study that assessed the efficacy of Office Hysteroscopy, 45 % of women were in premenopause and 55 % of them were in postmenopause. Regarding parity, 28 % were nulliparous or never had a vaginal delivery and 72 % had at least one vaginal delivery. Procedures were performed by both experienced hysteroscopists and trainees and were chirurgical in 239 cases, mainly polypectomies (191), followed by myomectomies (29), tubal ligation by Essure^®^ (24) and adhesion lysis (3). We performed 87 diagnostic hysteroscopies.

Of the total 330 hysteroscopies, only 23 % had HA. Fifty-six per cent of those patients who received HA referred good tolerance to the procedure, 37 % had a level of pain between 7 and 10, on a 0–10 scale, and in 7 %, the procedure was not concluded.

In this study, we concluded that OH was successful in about 92–95 % of the cases [9], without resorting to the OR. The procedures were generally well tolerated. In 330 cases by “see and treat” OH, 97.85 % [12] of the cases were successfully concluded and only 2.15% [9] of the patients were sent to the OR.

In the study that evaluated effectiveness of HA, patients had ages between 14 and 91 years (average 54); 95 of them were in premenopause and 112 in post menopause [12]. Regarding parity, 49 were nulliparous or never had a vaginal delivery and 158 had at least one vaginal delivery [12]. Procedures were performed by both experienced hysteroscopists and trainees and were chirurgical in 148 cases, mainly polypectomies (119), followed by myomectomies (15), tubal ligation by Essure® (15) and adhesion lysis (1) [12]. Only 59 hysteroscopies were diagnostic [12].

We concluded the procedure in 85 % [12] of patients, with mild to moderate pain referred. When HA was administered, in 54 cases [12], the intensity of pain was clearly inferior, allowing procedure closure in 93 % [12] of these patients, which otherwise would be sent to OR. Therefore, it proved to be effective and more comfortable to the patient, since there is no need for the use of speculum or procedure interruption.

In the study that evaluated the dimensions of the excised masses, patients had ages between 14 and 91 years (average 57), 40 % of them were in premenopause and 60 % in postmenopause [13]. Regarding parity, 26 % were nulliparous or never had a vaginal delivery and 74 % had at least one vaginal delivery [13]. Procedures were performed by both experienced hysteroscopists and trainees and were all chirurgical, mainly polypectomy, followed by myomectomy [13]. The biggest polyp removed was 72 mm long, and the biggest myoma was 58 mm long [13].

Population was divided in three groups, considering removed masses size: group 1, masses measuring less than 2 cm (158 procedures); group 2, masses of 2 cm or larger and inferior to 5 cm (55 procedures); and group 3, masses of 5 cm or larger (17 procedures) [13].

Most patients reported only slight pain or discomfort during the procedure [13]. The pain level was ≤5 in 73.1 % [13] of patients. Only 17 % [13] requested HA and 10 % [13] expressed willingness for general anaesthesia, but only 7.4 % were sent to OR, 52 % of those belonging to group 1 [13].On univariate analysis, size or number of masses was not related to the intensity of pain felt, or with the willingness for general anaesthesia [13].

We concluded that the probability of more than one procedure per mass was higher with larger masses, being 20 % of patients submitted to more than one hysteroscopy. Masses smaller than 2 cm did not need more than two procedures, and one patient with a mass larger than 5 cm needed four procedures [13].

In this study, we were able to excise 17 masses larger than 5 cm [13], thus concluding that the removal of masses larger than 5 cm is practicable in OH.

In 92.6 % [13] of patients, we were able to avoid the risks associated with general or locoregional anaesthesia as well as the adverse effects and costs associated to hospitalization.

## Conclusions

Office Hysteroscopy is a safe and effective technique, independent of the hospital circuit, not requiring the use of OR, hospital admission and general or locoregional anaesthesia, being therefore an important contribution for Patient Safety and Health Quality in gynaecology. Actually, OH has the ideal conditions to be qualified as the gold standard technique for the surgical treatment of intracavitary uterine pathology.

HA guaranties more comfort to the patient because it is speculum free and needs no hysteroscopy interruption. The use of HA, only when solicited, reduces costs and risks, effectively reducing pain, which allows closure of the procedure. Its focal characteristic also enhance HO capability, especially in myomectomies, where it can be administrated intracavitarily, preventing posterior OR procedure.

The removal of masses larger than 5 cm is controversial, but is possible, although it might imply more procedures per mass. That factor does not limit OH capability since the intensity of pain felt, or the willingness for general anaesthesia, is not directly related to the number of procedures.

It is therefore feasible to remove these masses, although it is technically challenging for the experienced hysteroscopist, because it implies balancing the tolerability of the patient and the control of the uterine cavity.
